# Research progress on long non-coding RNAs and their roles as potential biomarkers for diagnosis and prognosis in pancreatic cancer

**DOI:** 10.1186/s12935-020-01550-y

**Published:** 2020-09-15

**Authors:** Yizhi Wang, Li Zhou, Jun Lu, Bolun Jiang, Chengxi Liu, Junchao Guo, Gary Guishan Xiao

**Affiliations:** 1 Department of General Surgery, Peking Union Medical College Hospital, Chinese Academy of Medical Sciences and Peking Union Medical College, Beijing, 100730 China; 2 Pharmacy in Chemical Engineering School, Dalian University of Technology, Dalian, 116024 China; 3 Center for Functional Genomics and Proteomics, Creighton University Medical Center, Omaha, 68131 NE USA

**Keywords:** Pancreatic cancer, LncRNAs, Targeted therapy, Diagnosis, Prognosis

## Abstract

Pancreatic cancer is one of the main causes of tumor-related deaths worldwide because of its low morbidity but extremely high mortality, and is therefore colloquially known as the “king of cancer.” Sudden onset and lack of early diagnostic biomarkers directly contribute to the extremely high mortality rate of pancreatic cancer patients, and also make it indistinguishable from benign pancreatic diseases and precancerous pancreatic lesions. Additionally, the lack of effective prognostic biomarkers makes it difficult for clinicians to formulate precise follow-up strategies based on the postoperative characteristics of the patients, which results in missed early diagnosis of recurrent pancreatic cancer. Long non-coding RNAs (lncRNAs) can influence cell proliferation, invasion/migration, apoptosis, and even chemoresistance via regulation of various signaling pathways, leading to pro- or anti-cancer outcomes. Given the versatile effects of lncRNAs on tumor progression, using a single lncRNA or combination of several lncRNAs may be an effective method for tumor diagnosis and prognostic predictions. This review will give a comprehensive overview of the most recent research related to lncRNAs in pancreatic cancer progression, as targeted therapies, and as biomarkers for the diagnosis and prognosis of pancreatic cancer.

## Background

Pancreatic cancer tumors have some of the highest mortality rates worldwide, and the morbidity of this disease has not significantly declined [1]. The current 5-year overall survival (OS) rate of pancreatic cancer patients is estimated to be approximately 7%–9%. This OS rate even drops to a dismal 3% when distant metastases occur [2]. Pancreatic cancer is the fourth leading cause of cancer-related deaths with 47,050 dying from this disease in the United States, and is expected to take second place in 2030, just behind lung cancer [3]. In China, pancreatic cancer is the sixth leading cancer-related cause of death, and younger individuals have become increasingly threatened [4]. Difficult early diagnosis and lack of accurate prognostic biomarkers make it challenging to intervene in the early stages of initial diagnosis or of postoperative recurrent pancreatic cancer. This may cause unintended overdiagnosis and over-treatment to some extent, which is a major reason for the high mortality rate of pancreatic cancer [5–8].

Currently, computed tomography, magnetic resonance imaging, endoscopic ultrasound, and other imaging methods are used in the diagnosis and prognosis of pancreatic cancer. In addition, numerous serum biomarkers, such as circulating tumor DNA and certain microRNAs (miRNAs), can to some degree be used in these processes [9–11]. However, the relatively low specificity and sensitivity of these methods limit their clinical application in pancreatic cancer diagnosis and prognosis [12]. Therefore, novel biomarkers are urgently needed to increase the accuracy of early detection and treatment of this disease.

Long non-coding RNAs (lncRNAs) belong to a group of RNAs that are longer than 200 nucleotides and are not translated into proteins. Although lncRNAs are widely distributed throughout cells, they were initially considered as a kind of transcriptional “garbage” generated by RNA polymerase II, and were not believed to regulate any biological behaviors [13]. Recently, lncRNAs have been found to have a wide range of functions in various cellular processes, including chromatin modifications, gene transcription, post-transcriptional modifications, post-translational modifications, and functional protein localization through regulation of many intracellular signaling pathways [14]. Because of the versatility of lncRNAs in cells, it has been hypothesized that they play important roles in cancers, either tumor-promoting or tumor-suppressing, through their involvement in various cancer-related signaling pathways [15]. Pancreatic cancer is among the cancers affected by lncRNAs via multiple mechanisms [16]. This review will provide a comprehensive overview of these lncRNA-mediated mechanisms, potential lncRNA targeted therapies, and a discussion of the prospective diagnostic and prognostic value of these molecules in pancreatic cancer.

### Underlying mechanisms of lncRNAs in cancer

Since their discovery, lncRNAs have been implicated in the regulation of transcription, translation, and even post-translational modification of genes in both the nucleus and cytoplasm by binding to various RNAs or proteins [17]. In the nucleus, lncRNAs can interact with the components of chromatin-modifying complexes to guide them to the transcriptional regulatory regions of specific genes. This affects the structure of histones, which leads to altered expression of downstream genes [18]. For example, the lncRNA Xist can recruit polycomb repressive complex 2 (PRC2) to form Xist-PRC2 complexes to target specific X chromosome inactivation sites, where they catalyze the trimethylation of histone H3 lysine K27 (H3K27), a gene silencing histone modification [19]. Li et al. also revealed that lncRNA NMR can bind to chromatin regulator bromodomain PHD finger transcription factor to form a complex and activate the extracellular regulated protein kinases 1/2 (Erk 1/2) signaling pathway to promote esophageal squamous cell carcinoma progression [20]. Transcription factors can also bind to lncRNAs to modulate the transcription of downstream genes. In gastric cancer, overexpressed lncRNA AC093818.1 can bind to transcription factors signal transducer and activator of transcription 3 and Sp1 to increase the expression of 3-phosphoinositide-dependent protein kinase 1, phosphorylated protein kinase B, and phosphorylated mammalian target of rapamycin. This enhances the metastatic ability of gastric cancer cells [21]. Additionally, lncRNAs in the nucleus can bind to RNA binding proteins (RBPs) to regulate intracellular biological behaviors. Long intergenic noncoding RNA 01,413 (LINC01413) can bind to heterogeneous nuclear ribonucleoprotein-K and induce the nuclear translocation of yes-associated protein 1 (YAP1)/transcriptional adapter zinc binding 1 complex and upregulate the expression of Zinc Finger E-Box Binding Homeobox 1. This results in increased cell proliferation and promotes epithelial-mesenchymal transition [22]. Furthermore, Chang et al. indicated that LINC00997 can bind directly to the S100A11 promoter to enhance the invasion and migration ability of renal clear cell carcinoma cells [23].

**Fig. 1:**
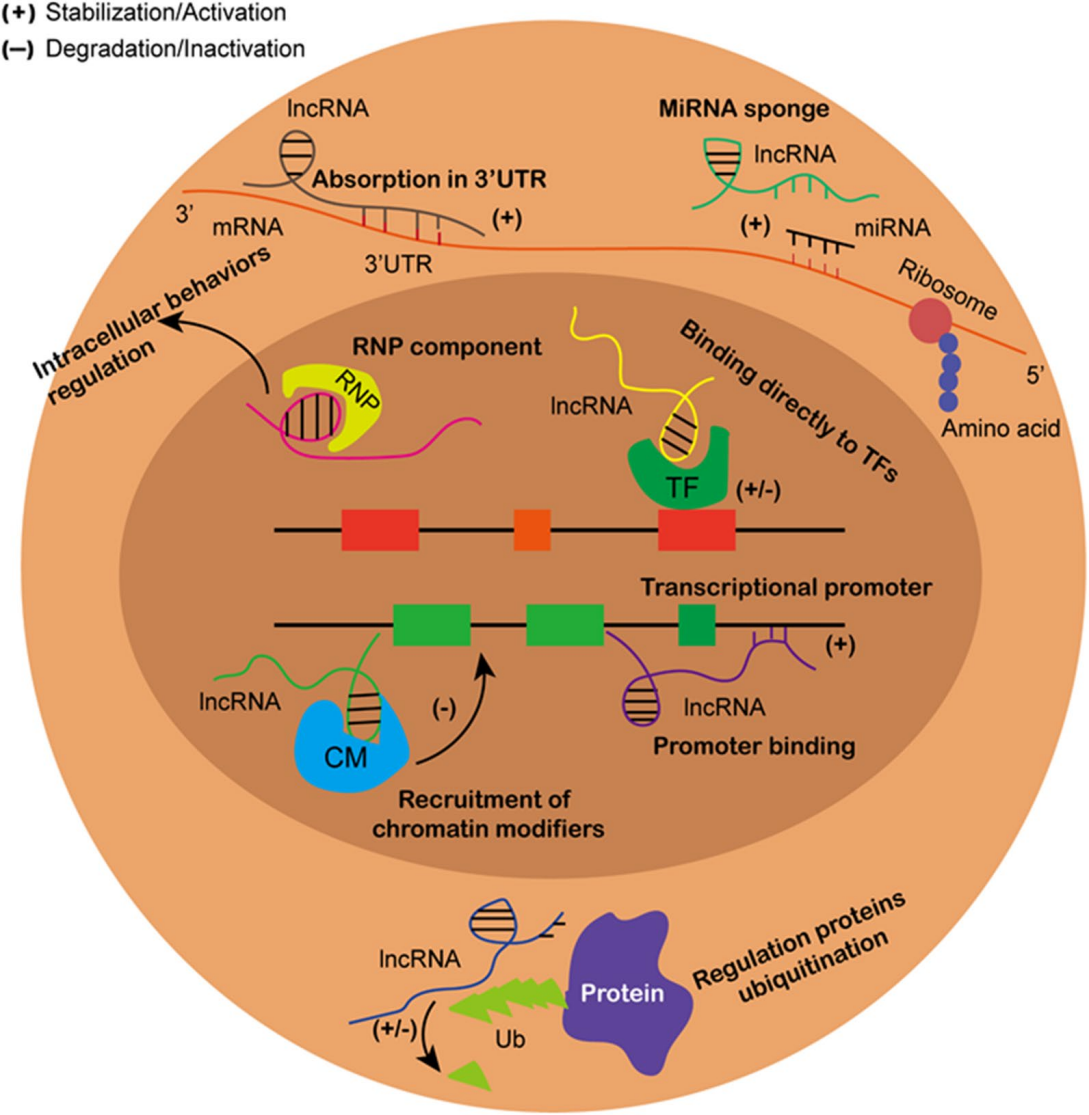
The mechanisms of lncRNAs in cancer progression. In nucleus, lncRNAs can recruit chromatin modifiers to bind to and effect targeted gene expression. LncRNAs can also bind directly to transcriptional factors and transcriptional promoters in nucleus regulating downstream gene expression. Besides, lncRNAs binding to RNA binding proteins can also influence a series of biological behaviors of tumor cells. In cytoplasm, lncRNAs and miRNAs competitively bind to mRNA which modulates targeted protein expression. LncRNAs can also directly bind to mRNAs to enhance their expression. Moreover, proteins ubiquitination can be affected by lncRNAs binding to targeted proteins

In the cytoplasm, the most well-established role of lncRNAs is acting as competing endogenous RNAs (ceRNAs) with miRNAs, thereby influencing mRNA expression that is regulated by the miRNAs [24]. MiRNAs exert their functions through binding of their 5′-end seed sequence with the 3′ untranslated region (3′ UTR) of target mRNAs, which inhibits protein expression of these genes [25]. LncRNAs can have multiple binding sites for certain miRNAs, which are completely or partially complementary to the corresponding sequences of the miRNAs. The lncRNAs can therefore act as a “sponge” and compete with the endogenous target genes for miRNA binding, thereby regulating the downstream mRNA expression levels. This mechanism forms a lncRNA-miRNA-mRNA regulatory network in many cancers [26–28]. In addition to interacting with miRNAs, cytoplasmic lncRNAs can directly bind to mRNAs to stabilize their expression. In an osteosarcoma study, Wu et al. showed that lncRNA THOR can bind directly to the middle region of the 3′ UTR of SRY-box 9 mRNA and enhance its expression, which subsequently increases the expression of aldehyde dehydrogenase 1 to enhance tumor cell stemness [29]. Post-translational modification is an important protein modification that can change the expression levels of specific proteins without modulating its mRNA expression levels. Ubiquitination is a common post-translational modification [30], and several studies have indicated that lncRNAs can play a role in this process in cancers. LncRNA ANCR can directly target Forkhead protein O1 and promote its ubiquitination and degradation without influencing its mRNA level, which facilitates gastric cancer progression [31]. Conversely, insulin-like growth factor 2 mRNA-binding protein 2 (IGF2BP2) was found to be downregulated in colorectal cancer. LncRNA LINRIS maintains IGF2BP2 stability by blocking ubiquitination of this protein at K139 [32]. The detailed mechanisms of lncRNAs in cancer progression are shown in Fig. 1.

### Roles of lncRNAs in pancreatic cancer

#### MiRNA sponge

LncRNAs can act as miRNA sponges and form lncRNA-miRNA-mRNA regulation networks to modulate the translation of mRNAs in pancreatic cancer [33]. According to the cancer-related role of each specifically affected mRNA, lncRNAs can be categorized as either tumor-promoting or tumor-suppressive. LncRNA FEZ family zinc finger 1 antisense RNA 1 (FEZF1-AS1) and zinc finger protein 312B (ZNF312B) were found to be upregulated in pancreatic cancer. FEZF1-AS1 overexpression enhanced tumor cell proliferation rates and invasion/migration ability, as well as the cell glycolytic capacity, through competitive binding of miR-107. Sponging of this miRNA resulted in increased expression of oncoprotein ZNF312B, a miR-107 target gene [34]. The Wnt/β-catenin signaling pathway plays a vital role in pancreatic cancer progression [35]. LncRNA BRAF-activated noncoding RNA was reported to competitively bind to miR-195-5p, promoting the expression of β-catenin and activation of Wnt/β-catenin signaling. This can in turn promote tumorigenesis [36]. LINC01559 was believed to play various roles in modulation of the YAP signaling pathways in the cytoplasm of pancreatic cancer cells [37]. In one mechanism, LINC01559 can act as a miRNA sponge of miR-607 to upregulate the expression of YAP1. In an additional mechanism, LINC01559 can also directly bind to YAP1 and block its phosphorylation, thereby promoting its activation [37]. A similar role was also discovered for lncRNA THAP9 antisense RNA 1, which not only sponges miR-484, but also directly binds to YAP1 and inhibits phosphorylation-mediated inactivation by large tumor suppressor gene 1. This then facilitates pancreatic cancer growth [38]. In addition to glycometabolism, lncRNAs participate in lipogenesis of tumor cells. Using Oil Red O staining analysis, Yu and colleagues found that knockdown of lncRNA Small nucleolar RNA host gene 16 (SNHG16) can block lipogenesis and inhibit the progression of pancreatic cancer. In-depth experiments showed that SNHG16 can sequester miR-195 and increase sterol regulatory element-binding protein 2 expression to achieve this biological function [39].

Other lncRNAs demonstrate tumor-suppressive roles through interactions with miRNAs. Exogenous administration of lncRNA growth arrest–specific transcript 5 (GAS5) can positively regulate the expression of phosphatase and tensin homologue through sponging of miR-32-5p to reduce tumor growth [40]. Pan et al. indicated that LINC01111 can upregulate Dual specificity phosphatase 1 expression by miR-3924 sequestration to block stress-activated protein kinase (SAPK) phosphorylation and inactivate the SAPK/c-Jun N-terminal kinase signaling pathway to suppress pancreatic cancer aggressiveness [41]. LINC00673 was also found to play a tumor-suppressive role through a miR-504/hepatocyte nuclear factor 1A axis [42]. Some examples of lncRNAs decoying miRNAs in pancreatic cancer are listed in Table 1.

**Table 1:** Some examples of lncRNAs decoying miRNAs in pancreatic cancer

LncRNA	Expression	Target of LncRNA	miRNA function	References
FEZF1-AS1	Up	miR-107	Targets ZNF312B	[34]
BANCR	Up	miR-195-5p	Regulate Wnt/β-catenin signaling pathway	[36]
LINC01559	Up	miR-607	Targets YAP	[37]
THAP9-AS1	Up	miR-484	Targets YAP	[38]
SNHG16	Up	miR-195	Targets SREBP2	[39]
LncRNA 00976	Up	miR-137	Targets OTUD7B	[43]
DIO3OS	Up	miR-122	Targets ALDOA	[44]
GAS5	Down	miR-32-5p	Targets PTEN	[40]
LINC01111	Down	miR-3924	Targets DUSP1	[41]
LINC00673	Down	miR-504	Targets HNF1A	[42]
PXN-AS1	Down	miR-3064	Targets PIP4K2B	[45]

FEZF1-AS1, FEZfamily zinc finger 1 antisense RNA 1; ZNF312B, zinc finger protein 312B; BANCR, BRAF-activated noncoding RNA; YAP, Yes-associated protein; THAP9-AS1, THAP9 antisense RNA 1; SNHG: Small nucleolar RNA host gene; SREBP, Sterol regulatory element-binding protein; OTUD7B, Ovarian tumor deubiquitinase 7B; DIO3OS, D3 gene opposite DNA strand; ALDOA, Aldolase, Fructose-Bisphosphate A; GAS5, growth arrest–specific transcript 5; PTEN, phosphatase and tensin homologue; DUSP, Dual specificity phosphatase; HNF1A, Hepatocyte nuclear factor 1A; PIP4K2B, phosphatidylinositol-4-phosphate 4-kinases 2B

### Chromatin remodeling

PRC2 is a chromatin-modifying complex that is composed of enhancer of zeste homolog 2 (EZH2), suppressor of zeste 12 and embryonic ectoderm development. PRC2 possesses methyltransferase activity to dimethylate or trimethylate lysine residue 27 of histone 3 (H3K27), which inhibits expression of downstream target genes [46]. Previous studies have revealed that about 20% of known lncRNAs can recruit and interact with components of PRC2 to enhance specific gene silencing, which could explain the aberrant expression of PRC2 observed in various cancers [47, 48]. Numerous lncRNAs can influence tumor progression via binding to PRC2. Hui et al. indicated that overexpression of lncRNA AGAP2-AS1 can promote the proliferation and metastasis of pancreatic cancer cells through recruitment of EZH2 and acting as a scaffold for the formation of PRC2. This leads to trimethylation of H3K27, which then inhibits the expression of Ankyrin repeat protein 1 and Angiopoietin-like 4 [49]. Wang et al. found that lncRNA SNHG15 was capable of binding to EZH2 and guiding it to the promoter regions of P15 and Kruppel-like factor 2 (KLF2), which induced H3K27 trimethylation to inhibit the expression of these two genes [50]. Subsequently, the same team also uncovered the underlying mechanism of lncRNA Taurine upregulated 1 (TUG1), which recruited EZH2 to the promoters of Rho family GTPase 3 and Metallothionein 2A, leading to inhibition of their transcription [51]. In addition to EZH2, some lncRNAs (DUXAP10, IRAIN, HOXA-AS1, and others) can bind to other chromatin modifiers, such as histone demethylase lysine-specific demethylase 1, to inhibit expression of downstream target genes [52–54]. Some examples of lncRNAs that interact with histone modifiers in pancreatic cancer are shown in Table 2.

**Table 2:** LncRNAs interacting with histone modifiers in pancreatic cancer

LncRNA	Molecule(s) interacting with lncRNA	Affected molecules	Epigenetic regulation caused by lncRNA on target genes	References
AGAP2-AS1	EZH2	ANKRD1 and ANGPTL4	Silencing of ANKRD1 and ANGPTL4	[49]
SNHG15	EZH2	P15 and KLF2	Inhibition of P15 and KLF2	[50]
TUG1	EZH2	RND3 and MT2A	Repressing of RND3 and MT2A	[51]
DUXAP10	EZH2 and LSD1	SMAD4 and KLF2	Silencing of SMAD4 and KLF2	[52]
IRAIN	EZH2 and LSD1	P15 and KLF2	Silencing of KLF2 and P15	[53]
HOXA-AS1	EZH2 and LSD1	–	–	[54]
HOTAIR	EZH2	DR5	Decrease the expression of DR5	[55]
SNHG14	EZH2	E-cadherin	Silencing the expression of E-cadherin	[56]

EZH2, Enhancer of zeste homolog 2; ANKRD1, Ankyrin repeat protein 1; ANGPTL4, Angiopoietin-like 4; SNHG, Small nucleolar RNA host gene; KLF2, Kruppel-like factor 2; TUG1, Taurine upregulated 1; RND3, Rho family GTPase 3; MT2A, Metallothionein 2A; LSD1, Lysine demethylase 1; SMAD4, SMAD family member 4; IRAIN, IGF1R antisense imprinted non-protein coding RNA; HOXA-AS1, HOXA cluster antisense RNA 2; HOTAIR, HOX transcript antisense RNA; DR5, Death receptor 5

### Direct interactions with proteins

LncRNAs can also directly bind to certain RBPs that regulate protein stabilization or degradation and therefore influence tumor progression. LncRNA Metastasis Associated Lung Adenocarcinoma Transcript 1 (MALAT1) can bind to RBP HuR, and this MALAT1/HuR complex can interact with the 3′ UTR of T cell intracellular antigen (TIA-1) mRNA and inhibit its translation, resulting in stimulation of autophagy in tumor cells [57]. Moreover, lncRNAs have also been reported to modulate metabolic reprogramming through enhancing protein stabilization. Qi et al. indicated that lncRNA metastasis associated gene of colorectal cancer 1-antisense RNA 1 (MACC1-AS1) facilitated aerobic glycolysis and activated the NOTCH signaling pathway by binding to and stabilizing pyruvate kinase M2 (PKM2) [58]. Ubiquitination is also involved in modulation of protein stabilization and can be regulated by lncRNAs in pancreatic cancer. Fused in sarcoma/translocated in liposarcoma (FUS/TLS) is thought to modulate RNA metabolism and various cellular processes such as proliferation and cell cycle progression [59, 60]. LncRNA SOX2OT can promote the proliferation of pancreatic cancer cells by forming physical bonds with FUS, which decreases its expression at the protein level without changing its mRNA levels [61]. Conversely, lncRNA XLOC_006390 can block c-Myc ubiquitination to upregulate the expression of glutamate dehydrogenase 1, which modulates glutamate metabolism [62]. The ubiquitination of p53 is also reversed by lncRNA CF129 binding to p53 and E3 ligase Makorin Ring Finger Protein 1 [63]. Additionally, lncRNA LINC01197 can bind to and segregate β-catenin and downstream T cell factor 4 without regulating β-catenin expression at both the mRNA and protein levels, leading to inactivated Wnt/β-catenin signaling [64].

### Binding to gene promoters

LncRNA XLOC_000647 can generate a series of tumor-suppressing biological behaviors through direct binding to the NOD-like receptor family pyrin domain-containing 3 promoter to inhibit its transcription [65]. Conversely, lncRNA ENST00000480739 positively regulates osteosarcoma amplified-9 by elevating the H3K27 acetylation level in its promoter, and then lncRNA ENST00000480739 negatively modulates hypoxia-inducible factor-1α expression [66].

### LncRNAs and chemoresistance of pancreatic cancer

**Fig. 2:**
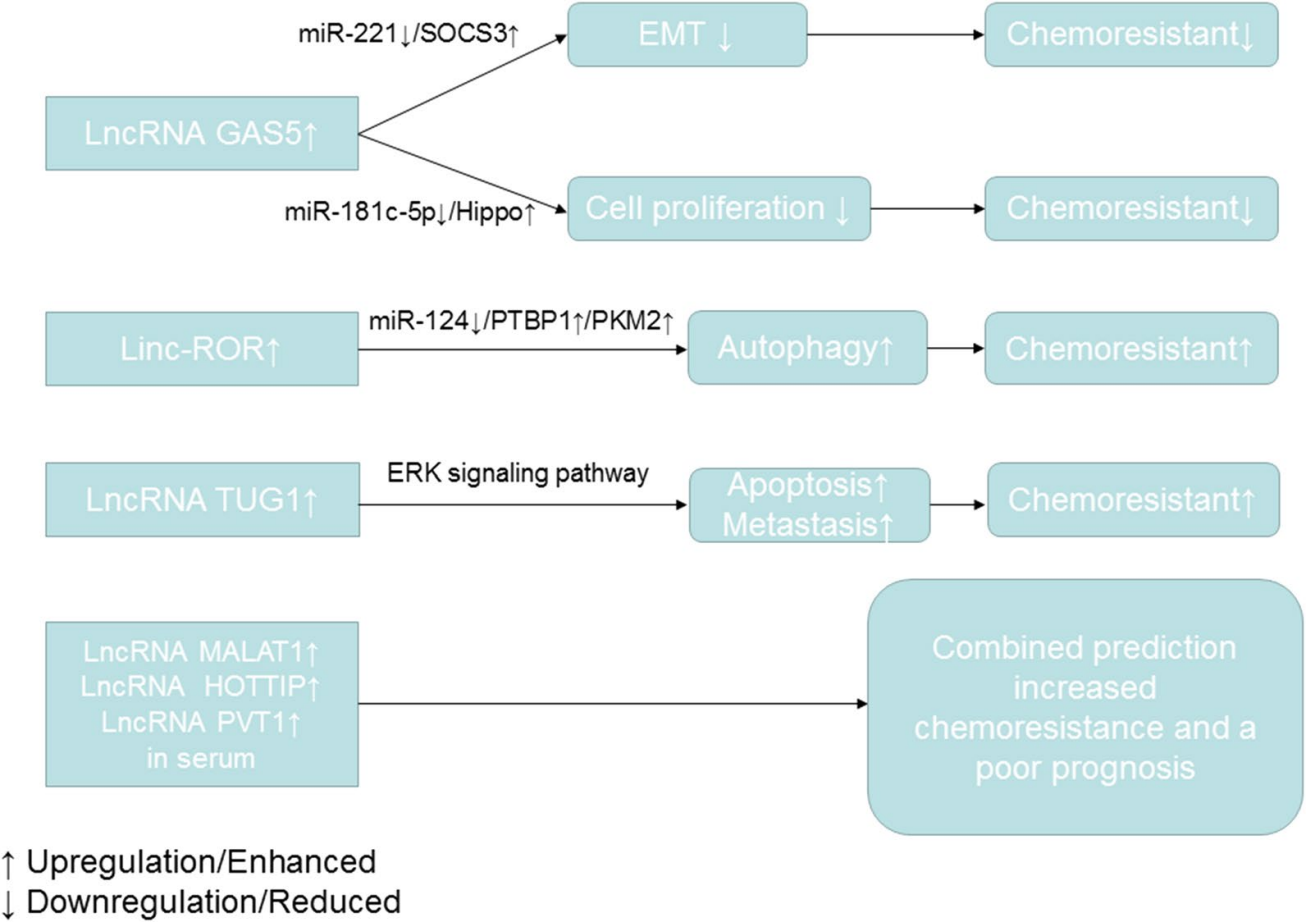
A diagram showing the roles of several lncRNAs in mediating chemoresistance and predicting chemotherapy response of pancreatic cancer

Chemoresistance is divided into two types: intrinsic and acquired [67, 68]. LncRNAs can reportedly modulate both intrinsic and acquired chemoresistance in pancreatic cancer through regulation of chemoresistance-related signaling pathways [69, 70]. Acting as a miRNA sponge is the main mechanism by which lncRNAs contribute to expression-induced chemoresistance. LncRNA GAS5 was downregulated in two pancreatic cancer chemoresistant cell lines, SW1990/gemcitabine and PATU8988/5-fluorouracil. MiR-181c-5p and miR-221 were involved in GAS5-induced chemoresistance through activating the Hippo signaling pathway and enhancing suppressor of cytokine signaling 3 expression [71, 72]. Upregulation of linc-ROR can mediate chemoresistance in pancreatic cancer cell lines via induction of autophagy. An in-depth study indicated that linc-ROR can bind to miR-124, which in turn enhanced the expression of polypyrimidine tract-binding protein 1 and promoted pyruvate kinase muscles 1 (PKM1) switching to PKM2 [73]. LncRNA TUG1-induced chemoresistance can be rescued by ERK pathway inhibitor SCH772984. Therefore, the ERK signaling pathway is implicated in TUG1-induced gemcitabine resistance. However, the detailed mechanisms controlling this still remain unknown [74]. Besides mediating chemoresistance, the level of specific lncRNAs in serum can predict the gemcitabine response rate of pancreatic cancer patients. Wang et al. found that higher levels of lncRNAs MALAT1, HOXA transcript at the distal tip, and plasmacytoma variant translocation 1 (PVT1) in the serum of pancreatic cancer patients correlated with lower patient response to gemcitabine-based chemotherapy and a shorter progression-free survival period [75]. Therefore, these lncRNAs may be used as prognostic biomarkers to estimate the therapeutic effect of gemcitabine in pancreatic cancer patients. A snapshot of the roles of lncRNAs in mediating chemoresistance of pancreatic cancer is shown in Fig. 2.

### LncRNAs as effective diagnostic and prognostic biomarkers in pancreatic cancer

Because of the various functions of lncRNAs in pancreatic cancer, detection of a single lncRNA or combination of several lncRNAs in body liquids or tumor tissues can be useful as biomarkers for early diagnosis or prognostic prediction of this disease [76]. LINC-PINT was found to be in lower abundance in plasma samples from pancreatic cancer patients than in those from healthy individuals. This lncRNA also had lower expression levels in pancreatic cancer tissues compared with tumors generated in adjacent tissues, such as the ampulla of Vater and common biliary duct tumors, indicating its better specificity. The diagnostic sensitivity of LINC-PINT is even stronger than that of CA19-9. Receiver operating characteristic curves showed that the area under the curve (AUC) of plasma LINC-PINT was 0.87, while that of CA19-9 was 0.78. The combination diagnostic sensitivity of LINC-PINT and CA19-9 is even stronger, with an AUC of 0.92 [77]. LncRNA SNHG15 may also be used as a diagnostic biomarker in pancreatic cancer because the levels of plasmatic SNHG15 were significantly higher in tumor patients than those in healthy individuals. The AUC was 0.785, showing a higher diagnostic potential [78]. Plasmatic LINC01638 has an even higher diagnostic sensitivity (AUC of 0.876) [79]. Through bioinformatic analysis, Liu et al. indicated that plasmatic lncRNA ABHD11-AS1 had a greater diagnostic sensitivity than CA19-9 (AUC of 0.887 and 0.78, respectively). The combination diagnostic sensitivity of ABHD11-AS1 and CA19-9 was also much higher (AUC of 0.982) [80]. Moreover, through analysis of the vesicle-encapsulated lncRNA HULC in plasma, the diagnostic efficiency of this lncRNA was determined to be better for distinguishing pancreatic cancer patients from healthy individuals than CA19-9 (AUC of 0.94 and 0.87, respectively). The diagnostic power of HULC for differentiating pancreatic cancer from intraductal papillary mucinous neoplasm (IPMN) was comparable to CA19-9 (AUC of 0.91 and 0.93, respectively) [81]. In a recent study, Yu et al. constructed a novel diagnostic (d-) signature comprising eight lncRNAs by detection of extracellular vesicle lncRNA profiling in the plasma of pancreatic cancer patients. The d-signature was able to identify resectable stage I/II pancreatic tumors with an AUC of 0.949. Additionally, this d-signature showed a better performance than CA19-9 in distinguishing pancreatic cancer from chronic pancreatitis (AUC of 0.931 and 0.873, respectively; *P *= 0.028) [82]. As well as in serum, lncRNAs detected in saliva can act as diagnostic biomarkers in pancreatic cancer. The lncRNAs HOTAIR and PVT1 had higher concentrations in saliva from pancreatic cancer patients than from healthy individuals, and their expression levels decreased after the patients underwent radical pancreatectomies. Therefore, HOTAIR and PVT1 detection in saliva may be used as a sensitive and noninvasive method to differentiate pancreatic cancer patients from healthy individuals or those with benign pancreatic lesions [83]. IPMN is believed to be precancerous lesions of pancreatic cancer, and it is necessary to distinguish malignant IPMN from benign IPMN so that radical operation can be performed in a timely manner to prolong the postoperative survival of patients [84]. Permuth et al. found that using a diagnostic model containing the plasmatic expression levels of an eight-lncRNA signature (including ADARB2-AS1, ANRIL, GLIS3-AS1, LINC00472, MEG3, PANDA, PVT1, and UCA1), worrisome features, gender, jaundice, radiomics, and a five-miRNA classifier signature can increase the diagnostic efficiency (AUC of 0.92) of distinguishing malignant IPMN from benign IPMN [85, 86].

Accurate prognostic prediction contributes to timely and specific therapeutic and follow-up strategies for pancreatic cancer patients who have undergone surgical treatment [87]. Numerous lncRNAs can be used as prognostic biomarkers in pancreatic cancer. LncRNA AFAP-AS1 was reported to have higher expression levels in tumor tissues than in para-tumor tissues, which indicated a worse prognosis with an AUC of 0.8669 when predicting progression within 1 year and 0.9370 when predicting progression within 6 months [88]. Bioinformatic analysis using TCGA (The Cancer Genome Atlas) database has established several lncRNA signatures to predict prognoses for pancreatic cancer patients postoperatively. Song et al. generated a prognostic analysis model containing five lncRNAs (including lncRNA C9orf139, MIR600HG, RP5-965G21.4, RP11-436K8.1, and CTC-327F10.4), which was considered to be an independent prognostic factor of OS. The AUC for this prediction model of 5-year OS was 0.742 [89]. Combinations of lncRNAs and miRNAs can also promote a better prognostic prediction efficiency. A panel of eight lncRNAs (including lncRNA RP3.470B24.5, CTA.941F9.9, RP11.557H15.3, LINC00960, AP000479.1, LINC00635, LINC00636, and AC073133.1) can make an effective prognostic prediction in pancreatic cancer patients with an AUC of 0.647. When combining these lncRNAs with an eight-mRNA signature (DHRS9, ONECUT1, OR8D4, MT1M, TCN1, MMP9, DPYSL3, and TTN), the AUC was raised to 0.716, increasing the prognostic prediction power of this model [90]. Although lncRNA-related prognostic models have a higher sensitivity based on bioinformatics, it is necessary to validate these results in a clinical cohort. Zhou et al. constructed a five-lncRNA signature (RP11-159F24.5, RP11-744N12.2, RP11-388M20.1, RP11-356C4.5, and CTC-459F4.9) and related it to OS using TCGA analysis. The authors then validated these results using a TCGA training cohort and an independent Fudan patient cohort. The prognostic prediction power of this lncRNA signature was stronger than using postoperative histological grade or TNM stage alone. The combination of this lncRNA signature with postoperative TNM stage resulted in an even better prognostic efficiency (AUC of 0.76 in the Fudan cohort) [91]. Examples of lncRNAs involved in pancreatic tumor diagnostic and prognostic prediction are listed in Table 3.

**Table 3:** LncRNAs involved in tumor diagnostic and prognostic prediction of pancreatic cancer

LncRNA	Diagnosis or prognosis	Sample	AUC	AUC when combined with non-lncRNA parameters	References
Linc-pint	Diagnosis (PDAC vs healthy control)	Plasma	0.87	0.92 (Combined with CA19-9)	[77]
SNHG15	Diagnosis (PDAC vs control)	Plasma/Tissues	0.727/0.785	–	[78]
LINC01638	Diagnosis (PDAC vs healthy control)	Plasma	0.8760	–	[79]
LncRNA ABHD11-AS1	Diagnosis (PDAC vs healthy control)	Plasma	0.887	0.982 (Combined with CA19-9)	[80]
LncRNA HULC	Diagnosis (PDAC vs healthy control)	Plasma (extracellular vesicle)	0.94	–	[81]
8-lncRNA signature (FGA, KRT19, HIST1H2BK, ITIH2, MARCH2, CLDN1, MAL2 and TIMP1)	Diagnosis (resectable PDAC vs healthy control)	Plasma (extracellular vesicle)	0.949	–	[82]
LncRNA HOTAIR and PVT1	Diagnosis (PDAC vs healthy control)	Salivary	0.909	0.870 (Combined with CA19-9)	[83]
	Diagnosis (PDAC vs benign pancreatic lesions)	Salivary	0.909	–	
	Diagnosis (PDAC vs IPMN)	Plasma (extracellular vesicle)	0.91	–	
8-lncRNA signature (ADARB2-AS1, ANRIL, GLIS3-AS1, LINC00472, MEG3, PANDA, PVT1 and UCA1)	Diagnosis (Malignant IPMN vs benign IPMN)	Plasma	0.77	0.9244 (A model combining the 8-lncRNA signature, the 5-miRNA signature, radiomic features, standard worrisome features (WF), gender, and presence of jaundice	[85]
LncRNA UFC1	Diagnosis (PDAC vs healthy control)	Plasma	0.810	–	[92]
LncRNA AFAP-AS1	Prognosis	Tissues	0.8669 (6 months)/0.9370 (1 year)	–	[88]
5-lncRNA signature (C9orf139, MIR600HG, RP5-965G21.4, RP11-436K8.1, and CTC-327F10.4)	Prognosis	Tissues	0.742	–	[89]
8-lncRNA signature (RP3.470B24.5, CTA.941F9.9, RP11.557H15.3, LINC00960, AP000479.1, LINC00635, LINC00636 and AC073133.1)	Prognosis	Tissues	0.647	0.716 (Combined a panel of 8-mRNA signature)	[90]
5-lncRNA signature (RP11-159F24.5, RP11-744N12.2, RP11-388M20.1, RP11-356C4.5, CTC-459F4.9)	Prognosis	Tissues	0.70	0.76 (Combined with TNM stage)	[91]
9-immune related lncRNA signature (AL138966.2, AL133520.1, AC142472.1, AC127024.5, AC116913.1, AC083880.1, AC124016.1, AC008443.5, and AC092171.5)	Prognosis	Tissues	0.703	–	[93]

AUC, Area under curve; PDAC, Pancreatic ductal adenocarcinoma; SNHG, Small nucleolar RNA host gene; CA, Carbohydrate antigen; ABHD11-AS1, α,β-hydrolase domain-containing protein 11-antisense 1; HOTAIR, HOX transcript antisense RNA; PVT, plasmacytoma variant translocation; HULC, Highly upregulated in liver cancer; IPMN, Intraductal papillary mucinous neoplasm; ANRIL, Antisense noncoding RNA in the INK4 locus; MEG, Maternally expressed

### Therapeutic potential of lncRNAs in pancreatic cancer

There are very few clinical trials using lncRNAs as targeted molecules for pancreatic cancer treatment. BC-819 (DTA-H19) is a double-stranded DNA plasmid carrying the diphtheria toxin A gene under the regulation of the H19 gene promoter. BC-819 can be concentrated most in tumor tissues that upregulate lncRNA H19 rather than in para-tumor tissues, and then subsequent transcription of the diphtheria toxin A gene can generate a cell killing effect. H19 is widely overexpressed in several cancer types, including pancreatic cancer [94]. Therefore, BC-819 has also been shown to have excellent tumor damaging effects in several cancers [95]. In a phase I/IIa clinical trial, intratumoral administration of BC-819 at a dose of 8 mg twice per week was confirmed to be safe and effective in patients with unresectable, locally advanced, non-metastatic pancreatic cancer. When combined with systemic chemotherapy, the therapeutic effect was even better [96].

In addition to the abovementioned tumor killing strategy, the expression, structure, and functions of lncRNAs in cancer cells can also be explored for therapeutic purposes. RNA interference technology, antisense oligonucleotides, or small molecule inhibitors can be used to inhibit lncRNA expression. Some small molecule inhibitors have been employed in several preclinical trials, but have yet to be used in clinical trials [97, 98]. Additionally, some beneficial tumor-suppressive lncRNAs can be delivered into the human body using viral vectors, such as adenoviruses or retroviruses, or non-viral vectors such as polyplexes, lipoplexes, and peptide- or protein-based systems [99]. Several modifications have also been applied to promote selective, highly localized, and targeted accumulation in cancer tissues [100]. However, few clinical trials of these therapeutic methods in pancreatic cancer have been conducted. Therefore, future studies should further focus on these lncRNA-targeted therapeutics to produce more efficacious drugs for treatment of pancreatic cancer.

## Conclusions and future perspectives

As a relatively newly discovered class of non-coding RNA, lncRNAs and their aberrant expression patterns play various roles in the progression of cancers, including pancreatic cancer. LncRNAs can modulate cell proliferation, cell cycle progression, and the invasion and migration abilities of pancreatic cancer cells through regulation of various signaling pathways in both the nucleus and cytoplasm. Given the extensive distribution and relatively stable structure of these molecules, lncRNAs can be used as effective biomarkers for risk prediction, diagnosis, and prognosis. The abnormal expression levels of specific lncRNAs in pancreatic cancer can be used to distinguish pancreatic cancer patients from healthy individuals or from patients with other types of pancreatic lesions. Increased diagnostic efficiency can contribute to a more precise and timely therapeutic strategy for treatment of this disease. Differential expression of lncRNAs in pancreatic cancer tissues and para-tumor tissues can also be prognostic to some extent.

Although the mechanisms and the diagnostic and prognostic roles of lncRNAs in pancreatic cancer have been well elucidated, there are still some areas that require further investigation. First, additional lncRNAs and their potential mechanisms in pancreatic cancer should be discovered through a series of basic experiments combined with bioinformatics. Second, owing to the limitations of the sensitivity and specificity of a single lncRNA, the combination of several lncRNAs based on bioinformatics or with conventional diagnostic or prognostic parameters, such as CA19-9, TNM stage, and histological grade, may enhance the diagnostic or prognostic power of this model. However, the accuracy of using these lncRNA signatures should be further verified by validation in a clinical cohort. Finally, there have been few lncRNA-targeted drugs in pancreatic cancer, and even in cancer therapy in general. Therefore, it is necessary to explore more lncRNA-targeted drugs or lncRNA-related signaling pathway inhibitors for future clinical practice.

## Data Availability

Not applicable.

## Abbreviations

AUC: Area under curve
BANCR: BRAF-activated noncoding RNA
CA19-9: Carbohydrate antigen 19-9
CM: Chromatin modifiers
CT: Computed tomography
EMT: Epithelial–mesenchymal transition
EZH2: Enhancer of zeste homolog 2
FEZF1-AS1: FEZfamily zinc finger 1 antisense RNA 1
FUS/TLS: Fused in sarcoma/translocated in liposarcoma
GAS5: Growth arrest–specific transcript 5
H3K27: Histone H3 lysine K27
HOTTIP: HOXA transcript at the distal tip
IGF2BP2: Insulin-like growth factor 2 mRNA-binding protein 2
IPMN: Intraductal papillary mucinous neoplasm
KLF2: Kruppel-like factor 2
LINC: Long intergenic noncoding
LncRNAs: Long non-coding RNAs
MACC1-AS1: Metastasis associated gene of colorectal cancer 1-antisense RNA1
MALAT1: Metastasis associated lung adenocarcinoma transcript 1
MRI: Magnetic resonance imaging
PKM1: Pyruvate kinase muscles 1
PRC2: Polycomb repressive complex 2
PVT1: Plasmacytoma variant translocation 1
RBPs: RNA binding proteins
ROC: Receiver operating characteristic
SAPK: Stress-activated protein kinase
SNHG: Small nucleolar RNA host gene 16
SOX9: SRY-box 9
TCGA: The Cancer Genome Atlas
TF: Transcriptional factor
TIA-1: T cell intracellular antigen
THAP9-AS1: THAP9 antisense RNA 1
TUG1: Taurine upregulated 1
YAP1: Yes-associated protein 1
NF312B: Zinc finger protein 312B
